# Exposure to Tobacco Smoke and Beliefs About Smoking Among Nursing Students and Professionals in Spain

**DOI:** 10.1111/phn.70086

**Published:** 2026-02-13

**Authors:** Mario García‐Suárez, Beatriz Ordás‐Campos, Alicia Álvarez‐Robles, Saúl Pérez‐Núñez, Laura López‐López, Marcos Vázquez‐Díez, Juan Gómez‐Salgado, Daniel Fernández‐García

**Affiliations:** ^1^ Health Research Nursing Group (GREIS) University of Leon Leon Spain; ^2^ Hospital Universitario de León León Spain; ^3^ Department of Nursing and Physiotherapy Faculty of Health Sciences University of León León Spain; ^4^ Department of Sociology Social Work and Public Health Faculty of Labour Sciences University of Huelva Huelva Spain; ^5^ Graduate Program in Health and Safety Universidad Espíritu Santo Guayaquil Ecuador

**Keywords:** belief, nurse, nursing, students, tobacco use

## Abstract

**Introduction:**

Tobacco use and exposure to tobacco smoke are considered to be among the main risk factors in the development of diseases in both children and adults. The role of nurses in identifying a smoker and making them aware of the consequences of smoking to help them to quit is essential. The aim of this study was to analyze the number of students and professionals exposed to tobacco smoke and to determine their opinions on the role of professionals in the prevention and cessation of smoking.

**Methodology:**

A descriptive cross‐sectional study was carried out using an anonymous self‐administered questionnaire, previously validated, which was distributed to nursing students between March and June 2022 and to nursing professionals between January and March 2023.

**Results:**

The prevalence of smoking was found to be at 14.5% among nursing students and at 19.1% among nursing professionals. A total of 41.1% of the students and 19.4% of the professionals were continuously exposed to tobacco smoke. Furthermore, professionals showed little to no agreement with statements such as “Not contributing to smoky environments” as a reason to quit smoking, “Health professionals act as social role models,” and “Smokers pay close attention to the recommendations made by health professionals” with respect to students (*p* < 0.001, *p* = 0.003, *p* = 0.018, respectively).

**Conclusions:**

An association was found between tobacco use and exposure to environmental tobacco smoke, showing that the creation and maintenance of smoke‐free spaces is essential. In addition, smoking students and professionals were found to be less inclined to provide smoking cessation advice to their patients. They further expressed a desire for further training to equip them with the necessary skills to offer such guidance.

## 1 Introduction

Tobacco use is the leading worldwide preventable cause of mortality, morbidity, and premature death, and therefore constitutes a serious threat to global health (World Health Organization 2022).

According to the Global Burden Disease (GBD) study published in 2021, the global prevalence of tobacco use had once again increased in most developed countries with respect to previous data, standing at 22.3% of the general population in 2020, with an age‐adjusted prevalence by sex of 36.7% and 7.8%, among men and women, respectively. These percentages by sex increased by 4% in the case of men and by 1.2% in the case of women during that year with respect to the previous one (Reitsma et al. 2021).

Moreover, knowledge of the number of people exposed to tobacco smoke (passive smoking), that is, involuntary exposure to tobacco smoke that does not result from active tobacco use, is particularly relevant. In 2006, the US Surgeon General's report coined the term “secondhand smoke” when referring to passive smoking. Secondhand smoke is composed of a mixture of smoke from the burning cigarette and smoke that is exhaled by the active smoker and returned back into the air (Pascual Lledó 2012; U.S. Department of Health and Human Services 2006). Exposure to secondhand smoke from this source is a cause of both premature mortality and disease in children and non‐smoking adults. In 2016, the GBD study estimated that, globally, exposure to secondhand smoke accounted for about 900,000 premature deaths (Gakidou et al. 2017; U.S. Department of Health and Human Services 2014).

In this regard, substantial progress has been made in many countries in limiting exposure to environmental tobacco smoke in public places, such as restaurants, bars, and entertainment venues. However, tobacco control laws do not apply in private spaces, such as private cars (though this is currently under consideration), or at home, where non‐smokers, including children, are exposed to tobacco smoke for long periods of time (Aurrekoetxea et al. 2016; Jones et al. 2014).

Additionally, the role of nurses in identifying the smoker is crucial, as well as in providing smoking cessation interventions through systematic counseling and patient follow‐up during the cessation process. Nurses, as a majority group among health professionals, hold a great potential to significantly contribute to the reduction of the tobacco epidemic in the community (Petersen et al. 2017; Rice et al. 2017).

Therefore, to ensure that nurses have the necessary knowledge, skills, and attitudes for effective smoking cessation interventions, it is necessary to provide adequate training in tobacco control (Petersen et al. 2017; Moxham et al. 2013; Malin et al. 2020; Sarna et al. 2015). In this respect, particularly relevant was the study by Bialous et al. which stressed the importance of training nurses in tobacco control, awareness of the health effects it can have on smokers, and the value of interventions for smoking cessation, which are essential for risks reduction (Bialous et al. 2017).

Therefore, the aim of this study was to analyze the exposure to tobacco smoke experienced by nursing students and professionals, as well as their opinions on the roles of health professionals in smoking prevention and the corresponding required training, in the city of Leon (Spain).

## 2 Methods

### 2.1 Design

A descriptive cross‐sectional study was designed using a self‐administered questionnaire, based on the recommendations of the STROBE (*Strengthening the Reporting of Observational Studies in Epidemiology*) statement (von Elm et al. 2007). The study was carried out at the Health Sciences School of the University of Leon and at the University Hospital of Leon, Spain. The research population consisted of students enrolled in the Nursing degree at the University of Leon and nursing professionals who were working at the University Hospital of Leon during the study period.

### 2.2 Participants

In the case of students, data collection was carried out between March and June 2022, with a total of 324 students participating out of the 400 enrolled (81% participation), far exceeding the required sample size for a confidence level of 95% and a margin of error of 5%. The inclusion criteria were to be enrolled in 1 of the 4 years of the Nursing degree and to be willing to fill in the questionnaire.

Regarding the professionals, data collection was carried out between January and March 2023, when a total of 252 nurses, out of the 1092 who on March 1, 2023 were working at the hospital (23.1% participation), decided to fill in the questionnaire and gave their informed consent. In this case, the required sample size (285 subjects) was not reached.

### 2.3 Instrument

The questionnaire was developed in accordance with the recommendations of the Regional Office for Europe of the World Health Organization (2002) and previously validated in a 2008 study (Martín et al. 2008), with additional amendments added in 2015 (Ordás et al. 2015) (Supporting Information File S1).

The sociodemographic variables collected included sex, age, academic year, and previous studies in the case of students. In the case of professionals, the collected variables were sex, age, type of contract, and years of professional experience.

Information was also collected on whether participants were “smokers,” if they reported using tobacco and/or e‐cigarettes, “ex‐smokers,” if participants reported having used tobacco and/or e‐cigarettes in the past but not currently, or “non‐smokers,” if participants reported never having used tobacco or e‐cigarettes. Participants who reported themselves as smokers were asked about the age of initiation of use, as well as about physical dependence on nicotine (Fagerström test) (Fagerstrom and Schneider 1989) and the motivation they had to quit smoking (Richmond Test) (Richmond et al. 1993).

The Fagerström test was used to measure physical dependence on nicotine, with values: 0–1: very low dependence; 2–3: low dependence; 4–5: moderate dependence; 6–7: high dependence; and 8–10: very high dependence. Motivation to quit smoking, which was categorized according to the Richmond test, was scored as: 0–4 points (low motivation); 5–6 points (moderate motivation); <7 points (high motivation).

In order to gain insight into participants’ attitudes and beliefs about smoking and smoking cessation, questions were asked about participants’ exposure to tobacco smoke in the week prior to the questionnaire and whether they were living with smokers at the time of the study.

As for their beliefs, participants were asked about the reasons for quitting smoking as health professionals, with response options being “Important” or “Not important.” Questions were also asked about the degree of agreement (“Agree” or “Disagree”) with a series of statements about the role of health professionals in smoking prevention, on the one hand, and about the training and behavior of professionals in relation to smoking, on the other. The items used in the questionnaire and answered by the participants are shown in the different tables in Section 3.

### 2.4 Statistical Analysis

A database was created using EpiInfo 7 software, which was also used for data analysis. A bivariate analysis was performed for continuous variables (age) using the *T*‐test if the data had a normal distribution and the Mann–Whitney *U*‐test otherwise. Homogeneity of variance was determined using Bartlett's test.

The chi‐squared test or Fisher's exact test (where necessary) were used for the bivariate analysis of categorical variables (sex, category, academic year, previous studies, type of contract, professional experience, and tobacco use). A value of *p* < 0.05 was considered statistically significant.

### 2.5 Ethical Aspects

This study followed the principles of the Declaration of Helsinki. This study was approved by an Ethics Committee of the University as well as by the Ethics Committee of the Hospital. The principles of confidentiality and the signing of the informed consent form were required for participation in the study. In addition, students were informed that not completing the questionnaire would not affect their academic progress.

## 3 Results

### 3.1 Sociodemographic Characteristics and Tobacco Use

A total of 576 participants completed the questionnaire, of which 324 (56.3%) were students and 252 (43.7%) were professionals, with a participation rate of 81% (324/400) and 23.1% (252/1092), respectively. Of the sample, 86.4% of the students were female, with a mean age of 21.2 (±5.2) years, and their previous studies were upper secondary studies. In the case of professionals, the female sex was predominant (215/252; 85.3%), with a mean age of 40.2 (±11.6) years and with more than 10 years of professional experience and a temporary contract. The rest of the sociodemographic characteristics can be found in Table 1.

**TABLE 1:** Sociodemographic characteristics of the participants and data on tobacco use.

			Socio‐demographic characteristics		Tobacco use		
			*n*	%	*n*	%	*p* value
Tobacco use	Students	Smokers			47	14.5	
		Ex‐smokers			22	6.8	
		Non‐smokers			255	78.7	
	Professionals	Smokers			48	19.1	
		Ex‐smokers			58	23	
		Non‐smokers			146	57.9	
Students	Sex	Female	280	86.4	42	15	0.524
		Male	44	13.6	5	11.4	
	Academic year	First	94	29	22	23.4	0.005^a^
		Second	90	27.8	15	16.7	
		Third	72	22.2	6	8.3	
		Fourth	68	21	4	5.9	
	Previous studies	Upper secondary	266	82.1	35	13.2	0.139
		Not upper secondary	58	17.9	12	20.7	
	Age	Mean ± SD	21.2 ± 5.2		15.7 ±1.5		
Professionals	Sex	Female	215	85.3	40	18.6	0.666
		Male	37	14.7	8	21.6	
	Work service	Specialized	94	37.3	15	16	0.601
		Hospitalization	118	46.8	24	20.3	
		Other	40	15.9	9	22.5	
	Type of contract	Permanent	114	45.2	17	14.9	0.129
		Temporary	138	54.8	31	22.5	
	Professional experience	>10 years	157	62.3	33	21	0.306
		˂10 years	95	37.7	15	15.8	
	Age	Mean ± SD	40.2 ± 11.6		17.8 ±3.1		

^a^ Post hoc analysis: statistical differences regarding tobacco use between first and third‐year students (23.4 vs. 8.33; *p* = 0.036) and first and fourth‐year students (23.4 vs. 5.88; *p* = 0.014).

Table 1 also provides the synthesized characteristics of tobacco use among the participants. Accordingly, the overall smoking prevalence amounted to 16.5% (95/576), being 14.5% (47/324) in students, and 19.1% (48/252) in nurses. More female students smoked than male students, but the situation was the other way around among professionals, and no significant differences were found in any of the cases. However, statistically significant differences were found between first‐year students and their peers, as they smoked more than third and fourth‐year students (*p* = 0.036 and *p* = 0.014), respectively.

Meanwhile, the age of smoking initiation in students was found to be 15.7 (±1.5) years and 17.8 (±3.1) years in professionals, this difference being statistically significant (*p* < 0.001). Regarding physical nicotine dependence, the Fagerström test showed a mean score of 2.73 points (SD = 2.25; minimum = 0; maximum = 8), indicating low physical nicotine dependence, with differences between professionals and students, as professionals had a slightly higher dependence (3.54 vs. 1.89; *p* < 0.001). Besides, motivation to quit smoking, as measured by the Richmond test, showed a mean score of 5.93 points (SD = 2.39; minimum = 0; maximum = 10), which indicated a moderate level of motivation to quit smoking among smokers, with no differences observed between professionals (6.1 points) and students (5.7 points).

### 3.2 Exposure to Environmental Tobacco Smoke and Living With Smokers

An assessment was made of which participants lived with smokers and which participants had been exposed to smoky environments on the days preceding the questionnaire. The results can be seen in Table 2.

**TABLE 2:** Participants living with smokers and participants exposed to environmental tobacco smoke at the time of the questionnaire.

			Living with smokers			Exposure to environmental tobacco smoke		
			*n*	%	*p* value	*n*	%	*p* value
Students	Sex	Female	120	42.9	0.808	115	41.1	0.791
		Male	18	40.9		19	43.2	
	Academic year	First	38	40.4	0.258	45	47.9	0.003^a^
		Second	34	37.8		44	48.9	
		Third	30	41.7		30	41.7	
		Fourth	36	52.9		15	22.1	
	Previous studies	Upper secondary	116	43.6	0.428	112	42.1	0.559
		Not upper secondary	22	37.9		22	37.9	
	Tobacco use	Smoker	24	51.1	0.204	32	68.1	<0.001
		Non‐smoker	114	41.2		102	36.8	
Professionals	Sex	Female	43	20	0.879	41	19.1	0.717
		Male	7	18.9		8	21.6	
	Service	Specialized	19	20.2	0.921	18	19.2	0.691
		Hospitalization	24	20.3		25	21.2	
		Other	7	17.5		6	15	
	Contract	Permanent	18	15.8	0.143	13	11.4	0.003
		Temporary	32	23.2		36	26.1	
	Professional experience	>10 years	25	15.9	0.045	18	11.5	<0.001
		˂10 years	25	26.3		31	32.6	
	Tobacco use	Smoker	17	35.4	0.003	18	37.5	<0.001
		Non‐smoker	33	16.2		31	15.2	
Comparison	Sex	Female	163	32.9	0.713	156	31.5	0.744
		Male	25	30.9		27	33.3	
	Category	Student	138	42.6	<0.001	134	41.4	<0.001
		Professional	50	19.8		49	19.4	
	Tobacco use	Smoker	41	43.2	0.016	50	52.6	<0.001
		Non‐smoker	147	30.6		133	27.7	

^a^ Post hoc analysis: statistical differences regarding exposure to tobacco smoke between first and fourth‐year students (*p* = 0.003), second and fourth‐year students (*p* = 0.003), and third and fourth‐year students (*p* = 0.036).

A total of 32.6% (188/576) of the respondents reported living with smokers, with students being more likely than professionals (*p* < 0.001) and smokers being more likely than non‐smokers to do so (*p* = 0.016). Similarly, 183 (31.8%) of the students and smokers said they had been more exposed to smoky environments than professionals and non‐smokers, with a significance level of *p* < 0.001 in both cases.

As just mentioned above, among the students, 42.6% (138/324) claimed to be living with smokers. On the other hand, 134 (41.4%) reported being exposed to smoky environments, with significant differences found in relation to the academic year, that is, first, second, and third‐year students being the most exposed to smoky environments. In addition, students who smoked were almost 3.7 times more likely to be exposed to smoky environments than non‐smokers (OR = 3.66; 95% CI: 1.89–7.08; *p* < 0.001).

Concerning the nursing professionals, 50 (19.8%) reported to be living with smokers. Of these, those with less than 10 years of experience who were tobacco users were more likely to live with smokers (*p* = 0.045 and *p* = 0.002). Furthermore, 19.4% (49/252) claimed to be exposed to tobacco smoke, with those with a temporary contract and less than 10 years of experience being the most exposed (*p* = 0.003 and *p* < 0.001). Similarly, smokers were more exposed to smoky environments than non‐smokers, as evidenced by the fact that smokers having almost 3.4 times the risk of being exposed to environmental tobacco smoke than non‐smokers (OR = 3.35; 95% CI: 1.67–6.73; *p* < 0.001).

### 3.3 Participants’ Opinions on the Reasons for Professionals to Quit Smoking

The results obtained with respect to this section can be seen in Table 3. In the group of students, differences were observed with regard to the reason “Setting a good example,” where smokers disagreed more than non‐smokers (27.7% vs. 15.2%; *p* = 0.035), with 83% of the participants considering it very important. Meanwhile, men considered “Not contributing to smoky environments” to be less important than women (13.6% vs. 3.9%; *p* = 0.007).

**TABLE 3:** Participants’ opinions on the reasons for professionals to quit smoking. Values expressed as *n* (%).

	Students			Professionals			Comparison		
	Important	Not important	*p* value	Important	Not important	*p* value	Important	Not important	*p* value
Protecting health	322 (99.4)	2 (0.6)		248 (98.4)	4 (1.6)		570 (99)	6 (1)	
Preventing tobacco‐related respiratory symptoms/diseases	323 (99.7)	1 (0.3)		249 (98.8)	3 (1.2)		572 (99.3)	4 (0.7)	
Pressure from other health professionals	205 (63.3)	119 (36.7)		145 (57.5)	107 (42.5)		350 (60.8)	226 (39.2)	
Setting a good example	269 (83)	55 (17)	0.004^a^ 0.035^b^	170 (67.5)	82 (32.5)		439 (76.2)	137 (23.8)	<0.001^d^
Not disturbing others nearby	298 (92)	26 (8)	0.028^a^	207 (82.1)	45 (17.9)	0.020^b^	505 (87.7)	71 (12.3)	0.026^c^ <0.001^d^
Not contributing to smoky environments	307 (94.8)	17 (5.2)	0.007^c^	205 (81.4)	47 (18.6)		512 (88.9)	64 (11.1)	0.002^c^ <0.001^d^

^a^ Differences by academic year.

Second and third‐year students believe that “Setting a good example” is of little or no importance compared to first‐year students (*p* = 0.009 and *p* = 0.016, respectively).

Second (*p* = 0.020), third (*p* = 0.015), and fourth‐year (*p* = 0.020) students considered ‘Not disturbing others nearby’ to be of little or no importance compared to first‐year students.

^b^ Differences by tobacco use.

^c^ Differences by sex.

^d^ Differences by category.

Among professionals, differences were found in “Not disturbing others nearby” as a reason to quit smoking, to which smokers attached more importance than non‐smokers (93.8% vs. 79.4%; *p* = 0.020).

Following data comparison, differences were found in the statements “Setting a good example,” “Not disturbing others nearby,” and “Not contributing to smoky environments,” where professionals considered these reasons to quit smoking to be of little or no importance, as compared to students (*p* < 0.001 in all cases). Similarly, men considered the reasons “Not disturbing others nearby” and “Not contributing to smoky environments” to be less important than women (*p* = 0.028; *p* = 0.002).

### 3.4 Participants’ Opinions on the Role of Professionals in Smoking Prevention and on the Training and Behavior of Professionals Regarding Tobacco Use

The results related to this section can be found in Table 4.

**TABLE 4:** Participants’ opinions on the role of health professionals in the prevention of smoking and on the training and behavior of professionals regarding smoking. Values expressed as *n* (%).

	Students			Professionals			Comparison		
	Agree	Disagree	*p* value	Agree	Disagree	*p* value	Agree	Disagree	*p* value
**Role of professionals**									
Professionals’ advice is very important in helping to quit smoking	301 (92.9)	23 (7.1)	0.028^a^	217 (86.1)	35 (13.9)		518 (89.9)	58 (10.1)	0.043^b^ 0.007^g^
Health professionals should never smoke in front of their patients to set a good example	288 (88.9)	36 (11.1)	<0.001^b^	231 (91.7)	21 (8.3)		519 (90.1)	57 (9.9)	<0.001^b^
Health professionals act as social role models in relation to the habit	294 (90.7)	30 (9.3)		190 (75.4)	62 (24.6)	0.002^b^ 0.045^e^	484 (84)	92 (16)	0.003^b^ <0.001^g^
Smokers pay close attention to the recommendations made by health professionals	162 (50)	162 (50)		101 (40.1)	151 (59.9)		263 (45.7)	313 (54.3)	0.018^g^
Health professionals have an obligation to try to convince patients to quit smoking	263 (81.2)	61 (18.8)		187 (74.2)	65 (25.8)	0.032^f^	450 (78.1)	126 (21.9)	0.045^g^
**Training and behavior**									
My knowledge allows me to report rigorously on the consequences of tobacco consumption	286 (88.3)	38 (11.7)		217 (86.1)	35 (13.9)		503 (87.3)	73 (12.7)	
I have sufficient knowledge to help patients to effectively quit smoking	175 (54)	149 (46)	<0.001^c^ 0.019^d^	133 (52.8)	119 (47.2)		308 (53.5)	268 (46.5)	0.005^c^
Students need specific training and education to help patients to quit smoking	313 (96.6)	11 (3.4)		239 (94.8)	13 (5.2)		552 (95.8)	24 (4.2)	
Issues related to smoking prevention should be included in the training	311 (96)	13 (4)		245 (97.2)	7 (2.8)		556 (96.5)	20 (3.5)	
Health professionals play a very important social role in preventing tobacco use	307 (94.8)	17 (5.2)		214 (84.9)	38 (15.1)		521 (90.5)	55 (9.5)	0.008^b^ <0.001^g^
I am aware of strategies and methods to help patients quit smoking	162 (50)	162 (50)	0.008^d^	128 (50.8)	124 (49.2)		290 (50.4)	286 (49.6)	

^a^ Differences by previous studies.

^b^ Differences by tobacco use.

^c^ Differences by sex.

^d^ Differences by academic year.

^e^ Differences by professional experience.

^f^ Differences by type of contract.

^g^ Differences by category.

Concerning the role of professionals in smoking prevention, students whose educational background was not upper secondary studies were less in agreement with the statement “Professionals’ advice is very important to help patients quit smoking” than those who did come from upper secondary studies (13.8% vs. 5.6%; *p* < 0.028). At the same time, less smokers agreed with the statement “Health professionals should never smoke in front of their patients to set a good example” (27.7% vs. 8.3%; *p* < 0.001), as compared to non‐smokers.

As for professionals, the most inexperienced and the smokers were in disagreement with those who had more than 10 years of experience (31.6% vs. 20.4%; *p* = 0.045) and non‐smokers (41.7% vs. 20.6%; *p* = 0.002) on the statement defining health professionals as social role models, and those with a temporary contract were also in disagreement on the statement “Health professionals have an obligation to try to convince patients to quit smoking” as compared to those with a permanent contract (31.2% vs. 19.3%; *p* = 0.032).

Finally, differences were found between professionals and smokers’ opinions and those of students and non‐smokers regarding the statements “Professionals’ advice is very important to help patients quit smoking” (13.9% vs. 7.1%; *p* = 0007) and (15.8% vs. 8.9%; *p* = 0.043), respectively, and “Health professionals act as social role models” (24.6% vs. 9.3%; *p* < 0.001) and (26.3% vs. 13.9%; *p* = 0.003). Furthermore, professionals also expressed disagreement on the statements “Health professionals have an obligation to try to convince patients to quit smoking” (25.8% vs. 18.8%; *p* = 0.045) and “Smokers pay close attention to the recommendations made by health professionals” (59.9% vs. 50%; *p* = 0.018), while smokers also disagreed on the statement “Health professionals should never smoke in front of their patients to set a good example” (20% vs. 7.9%; *p* < 0.001).

In terms of the opinions on the training and behavior of professionals, 88.3% of the students stated that they had sufficient knowledge to inform about the consequences of smoking, but differences were found in the statement “I have sufficient knowledge to help patients to effectively quit smoking,” in which men were more in agreement than women (77.3% vs. 50.4%; *p* < 0.001). There were also differences with respect to the academic year (*p* = 0.019) where, after the post hoc analysis, it was found that third‐year students were more likely to agree on this statement than first‐year students (66.7% vs. 42.6%; *p* < 0.016).

Similarly, differences were also found by academic year in the statement “I am aware of strategies and methods to help patients quit smoking” (*p* = 0.008), where students in the first year always expressed to be less aware than those in the second year (35.1% vs. 55.6%; *p* = 0.021), third year (35.1% vs. 56.9%; *p* = 0.021), and fourth year (35.1% vs. 55.9%; *p* = 0.021).

With regard to professionals, 47.2% and 49.2% of respondents expressed disagreement with the statements about having sufficient knowledge to help patients quit smoking and about being aware of strategies and methods to help patients quit smoking, respectively. Moreover, in this case, no statistical differences were found.

When comparing data, differences were observed in women (they were more likely to disagree than men) on “I have sufficient knowledge to help patients to effectively quit smoking” (48.9% vs. 32.1%; *p* = 0.005) and on “Health professionals play a very important social role in preventing tobacco use.” In this line, both professionals and smokers were more likely to disagree more than students (15.1% vs. 5.3%; *p* < 0.001) and non‐smokers (16.8% vs. 8.1%; *p* = 0.008).

## 4 Discussion

In the present study, exposure to tobacco smoke and living with smokers was assessed in nursing students and professionals, together with the different opinions on certain aspects such as training and the role of health professionals regarding tobacco consumption and its prevention.

The profile of the student participating in this study was female, in line with the literature reviewed (Moxham et al. 2013; Ordás et al. 2015; Martínez et al. 2021; Ortega‐Ceballos et al. 2018; Fernández et al. 2015; Saraiva et al. 2017; Pingak and Miller 2019; Provenzano et al. 2019), aged between 19 and 21 years, as in five of the reviewed studies (Ordás et al. 2015; Ortega‐Ceballos et al. 2018; Fernández et al. 2015; Saraiva et al. 2017; Provenzano et al. 2019), and accessing through upper secondary studies. The profile of the professional was also female, as in nine analyzed studies (Malin et al. 2020; Hodgetts et al. 2004; Chan et al. 2007; Pericás et al. 2007; O'Donovan 2009; Pérez Saavedra et al. 2010; Sarna et al. 2012; Sonmez et al. 2015; Lee et al. 2021), with a mean age between 30 and 50 years, which was also in line with the reviewed literature (Malin et al. 2020; Hodgetts et al. 2004; Pericás et al. 2007; Pérez Saavedra et al. 2010; Lee et al. 2021; Bloor et al. 2006).

As for tobacco use, its prevalence, as shown in the data, was at 14.5% for students and at 19.1% for professionals. In the case of students, data were much lower than those obtained in the review by Zeng et al. (2020), in which the prevalence of tobacco use among nursing students was at 26.6%. This is in line with studies carried out in Spain, which showed a prevalence of between 17.6% and 29.7% among student smokers (Ordás et al. 2015; Martínez et al. 2021; Fernández et al. 2015), and several studies published in Mexico (Ortega‐Ceballos et al. 2018), Portugal (Saraiva et al. 2017), Indonesia (Pingak and Miller 2019), and Italy (Provenzano et al. 2019), where a higher prevalence, ranging from 25% to 45%, was identified. Only the study by Moxham et al. (2013) showed a lower prevalence than in the present study, with 7.4% of students being active smokers. In this regard, it should be noted that the population in the present study was not statistically representative, so these results should be treated with caution.

The prevalence of tobacco use was also analyzed in a review conducted by Nilan et al. among professionals from various branches of the Health Sciences (Nilan et al. 2019), where a total of 229 studies were included. The study revealed a prevalence rate at 21%, indicating a decline in prevalence over the 15‐year period. Specifically, it decreased from 23% in 2000 to 18% in 2015. Notably, nurses consistently exhibited the highest rates of tobacco use. This is consistent with seven other studies reviewed in which the prevalence was higher than that shown in the present study (Hodgetts et al. 2004; Pericás et al. 2007; O'Donovan 2009; Sonmez et al. 2015; Bloor et al. 2006; Abou‐ElWafa et al. 2020; Hoseainrezaee et al. 2013). Besides, four other studies carried out in Finland (Malin et al. 2020), China (Chan et al. 2007), Peru (Pérez Saavedra et al. 2010), and the United States (Sarna et al. 2012) found a lower prevalence of tobacco use, ranging from 2.2% to 12%.

With regard to the age at which consumption began, both students and professionals started using tobacco in their school years and adolescence. In the case of students, the data can be compared to those from different studies carried out in Spain and Mexico, where the mean age at which students started smoking was around 15 and 17 years of age or even younger, and remained the same over the years (Moxham et al. 2013; Ordás et al. 2015; Ortega‐Ceballos et al. 2018; Fernández et al. 2015; Saraiva et al. 2017). Regarding professionals, comparison is more limited, as most of the studies identified 20 years as the starting age for tobacco use. Indeed, one study found that smoking started before the age of 20 (Abou‐ElWafa et al. 2020) and another two after that age (Pérez Saavedra et al. 2010; Sonmez et al. 2015). Only O'Donovan (2009), with a mean age of initiation at 16.1 years, gave an accurate value for the age at which participants started smoking.

In terms of physical nicotine dependence and motivation to quit smoking, in the case of students, a large variability was found to exist between the scales used to measure nicotine dependence; in spite of this, the majority of the articles reported a low mean dependence in all participants (Ordás et al. 2015; Fernández et al. 2015; Saraiva et al. 2017; Provenzano et al. 2019). In the case of professionals, the article by Sonmez et al. (2015) showed a low mean dependence rate among the participants, while a study in Egypt found that dependence was significantly higher among nurses when they were in the workplace (Abou‐ElWafa et al. 2020). In this case, no studies were identified that measured motivation to quit smoking, although some studies surveyed professionals about whether they were interested in quitting smoking and whether they would try to quit within a certain period of time, as well as their reasons for quitting (Hodgetts et al. 2004; Pericás et al. 2007; O'Donovan 2009; Sarna et al. 2012; Sonmez et al. 2015; Bloor et al. 2006; Hoseainrezaee et al. 2013).

With regard to the exposure of students to smoky environments, this variable was of high importance among smokers, although a high percentage of non‐smokers also attached great importance to it. Despite obtaining significant differences, the data are still relevant, as in Spain, in 2011, Law 42/2010 of 30 December was introduced, which amended Law 28/2005 of 26 December on health measures against smoking and regulating the sale, supply, consumption, and advertising of tobacco products (Boletín Oficial del Estado 2010), and a series of measures and restrictions on tobacco consumption were imposed, particularly in public places. Few studies referred to exposure to tobacco smoke and they did not differentiate between smokers and non‐smokers (Moxham et al. 2013; Ortega‐Ceballos et al. 2018: Saraiva et al. 2017; Provenzano et al. 2019). Despite this, the percentages obtained were also high, ranging from 44.5% in an Australian study (Moxham et al. 2013) to 59% in two studies, one carried out in Mexico and another in Italy (Ortega‐Ceballos et al. 2018; Provenzano et al. 2019). Additionally, the Mexican study also found that 27% of students were exposed to smoky environments at home (Ortega‐Ceballos et al. 2018). It is possible that in these countries there was not such a strict law regarding smoking in public places and/or enclosed spaces at the time of the study, and that, therefore, overall cigarette consumption was much higher, which accounts for the fact that the number of people exposed to tobacco smoke was even higher than in the present study. With regard to students who lived with smokers, the percentage in this study was similar to that obtained in the evidence consulted, with values between 45% and 60% (Moxham et al. 2013; Saraiva et al. 2017; Provenzano et al. 2019).

It is significant, too, that the professionals in the present study with the least years of experience and the worst type of contract were the most likely to live with smokers and to be exposed to tobacco smoke, since it is assumed that these are the professionals who have most recently completed their studies and in the vast majority of cases are the youngest in age. In the literature, the study by Pérez Saavedra et al. (2010) showed the importance of exposure to tobacco smoke in that 10% of professionals were exposed to smoky environments at home and 25% outside the home. Furthermore, Pericás et al. (2007) identified that 33% of the professionals surveyed were bothered by tobacco smoke in shared spaces and so more than half had never complained about anyone smoking in a shared space.

With regard to the participants’ opinions on the reasons for professionals to quit smoking, in the case of students, in only two studies did the majority of students considered it important to set a good example by not smoking in front of patients (Moxham et al. 2013; Ordás et al. 2015), which is in line with the results obtained in the present study. Among professionals, the results found in this respect were also in line with those obtained from the students, and differences were found with regard to the reason “Not disturbing others nearby,” to which smokers attached more importance than non‐smokers. This data can be compared with those from the study by Sonmez et al. (2015), in which 94% reported that smoking should be banned in places where it could disturb other people. In addition, an Australian and a UK study agreed at over 60% that the health of smokers and passive smokers should be protected specifically in the workplace (O'Donovan 2009; Bloor et al. 2006).

Concerning the opinions of the participants on the role of professionals in the prevention of smoking and the training and behavior of professionals in this respect, in this study, the data provided by the students significantly coincided except for the statement “Smokers pay close attention to the recommendations made by health professionals,” where they obtained a degree of disagreement at 63% (Fernández et al. 2015). In another study carried out over 10 years, these data were stable at around 64%, although one year the percentage dropped to 55% (Ordás et al. 2015). Meanwhile, the study by Martínez et al. (2021) showed that 56% of students believed that smokers were more likely to quit if advised by a professional, and the study by Moxham et al. (2013) found 70% and 67% agreement among students that professionals should advise smokers to quit and that the chances of quitting increased if the professional was the one giving the advice, respectively. Pingak and Miller (2019) reported that 96.5% of the students believed that professionals had to advise smokers on quitting smoking. Finally, Ortega‐Ceballos et al. (2018) reported that 73% of the students believed that the behavior displayed by the professional was taken as a role model for patients, a lower figure than the one obtained in this study.

In the case of professionals, up to eight studies included statements similar to those described in this section (Hodgetts et al. 2004; Pericás et al. 2007; Pérez Saavedra et al. 2010; Sarna et al. 2012; Sonmez et al. 2015; Lee et al. 2021; Bloor et al. 2006; Abou‐ElWafa et al. 2020). Regarding not smoking in front of patients in order to set a good example, five studies (Hodgetts et al. 2004; Pérez Saavedra et al. 2010; Sonmez et al. 2015; Lee et al. 2021; Abou‐ElWafa et al. 2020) showed agreement in more than 90% of cases, as in the present study, but Bloor et al. (2006) only found 39% agreement among professionals. Also, regarding the ability to convince or advise patients to quit smoking, mixed results were found as, in a study conducted in 2012 (Sarna et al. 2012), 30% of respondents disagreed for considering this intervention to be inefficient; conversely, other studies agreed in 60%–90% of cases (Pérez Saavedra et al. 2010; Bloor et al. 2006; Abou‐ElWafa et al. 2020). Regarding the definition of professionals as social role models, only Sarna et al. (2012) assessed this item, where 98% of their surveyed professionals agreed.

Finally, for questions referring to the training and behavior of health professionals regarding smoking cessation, in the case of students, several studies addressed the level of knowledge and training of students in helping patients to quit smoking (Ordás et al. 2015; Martínez et al. 2021; Ortega‐Ceballos et al. 2018; Fernández et al. 2015; Pingak and Miller 2019). Thus, according to the studies by Pingak and Miller (2019), Fernández et al. (2015), and Martínez et al. (2021), 85%, 47.8%, and 24%, respectively, of students indicated that they had sufficient knowledge to help patients quit smoking. In contrast, in Mexico, according to Ortega‐Ceballos et al. (2018), 87% of the students reported that they had not received any training of this type, but 97% considered that the professional should be trained in cessation techniques. In addition, the study by Martínez et al. (2021) found that 87% had received training on the risks of tobacco use, but only 33% of the students had received training on smoking cessation techniques.

In the case of professionals, a total of nine studies addressed smoking cessation training and cessation strategies (Malin et al. 2020; Chan et al. 2007; Pérez Saavedra et al. 2010; Sarna et al. 2012; Sonmez et al. 2015; Lee et al. 2021; Abou‐ElWafa et al. 2020; Hoseainrezaee et al. 2013). As to whether the professionals had received training in smoking cessation at some point, the participants in six studies stated so, with percentages ranging from 50% to 85.6% (Hodgetts et al. 2004; Chan et al. 2007; Pérez Saavedra et al. 2010; Sarna et al. 2012; Sonmez et al. 2015; Lee et al. 2021), and the study by Abou‐ElWafa et al. 2020 showed that 80% of professionals believed that training should also be aimed at professionals once they have finished their undergraduate studies. The social role in preventing consumption was also assessed in four articles, which showed strong agreement, with percentages above 80% (Pérez Saavedra et al. 2010; Sonmez et al. 2015; Lee et al. 2021; Abou‐ElWafa et al. 2020). Lastly, regarding the fact of being aware of strategies or having sufficient knowledge to help quit smoking, only three studies carried out in the United States (Sarna et al. 2012), Finland (Malin et al. 2020), and China (Chan et al. 2007) obtained percentages of 63%, 75%, and 89.3%, respectively, with the American and Chinese ones showing that 53% and 80% of their respondents informed their patients based on the knowledge they had acquired.

When analyzing in depth training and behavior in relation to smoking, a great deal of heterogeneity was found in the results obtained from students and professionals. Thus, it can be determined that the level of knowledge to help quit smoking was quite low in both groups compared to the level of knowledge of both groups to inform about the consequences of tobacco consumption.

As for the limitations of the study, it should be noted that no distinction was made between the type of consumption and its frequency, so that it was not determined whether smokers were daily or occasional smokers. Therefore, the values obtained in the tests to measure nicotine dependence and motivation to quit smoking could have been affected in their overall result.

Additionally, in relation to the answers concerning the different opinions of the participants, respondents may have introduced a social desirability bias toward the answer they considered socially acceptable as opposed to their own opinion. However, the anonymous nature of the questionnaire and the absence of an interviewer when the questionnaire was completed may have minimized this effect.

Finally, not having a statistically significant sample may have compromised the validity of the results.

## 5 Conclusions

The prevalence of tobacco use among nursing students and professionals obtained in this study was lower in the case of students and similar for professionals, as compared to the general population of the same age range.

An association was shown between tobacco consumption and exposure to environmental tobacco smoke, which justifies keeping as many environments and public spaces smoke‐free as possible, as this is an easy and cost‐effective measure that can prevent an increase in the incidence of smoking. In addition, students were more likely to live with smokers than professionals, thus having a higher exposure to tobacco smoke.

Both students and professionals who smoked found it difficult to provide anti‐tobacco health counselling. Although they agreed with many of the proposed statements, the participants showed low regard for their potential as health workers and reported wanting more training and information to be able to develop their roles as health professionals in this respect.

This line of research is to be continued by carrying out multicenter studies involving several Nursing schools and hospitals, using a significant sampling, and also considering the inclusion of Primary Care professionals, who are often responsible for community activities aimed at smoking cessation.

## 6 Clinical Relevance

*What is already known*

‐ The role of nurses in identifying smokers and raising awareness about the consequences of smoking to support smoking cessation is essential.
‐ To ensure that nurses possess the necessary knowledge, skills, and attitudes for effective smoking cessation interventions, appropriate training in tobacco control must be provided.

*What this paper adds*

‐ This study examines tobacco smoke exposure and perspectives on the role of healthcare professionals in smoking prevention and cessation among nursing students and professionals in León, Spain.
‐ It was found that smoking students and professionals were less inclined to provide smoking cessation advice and expressed a desire for additional training to fulfil their roles in this area.
‐ Professionals showed less agreement than students regarding statements about their role as social models and the importance of not contributing to smoke‐filled environments.

## 7 Clinical Resources

World Health Organization (WHO)—Tobacco Free Initiative https://www.who.int/health‐topics/tobacco Centers for Disease Control and Prevention (CDC)—Smoking & Tobacco Use https://www.cdc.gov/tobacco/

American Lung Association—Smoking Cessation Resources https://www.lung.org/quit‐smoking

National Cancer Institute—Smoking Cessation Resources https://www.cancer.gov/about‐cancer/causes‐prevention/risk/tobacco

Global Bridges—Healthcare Alliance for Tobacco Dependence Treatment https://www.globalbridges.org/

## Ethics Statement

This study was approved by the Ethics Committee of the University of Leon, with registration number: ETICA‐ULE‐030‐2022, as well as by the Ethics Committee of the Hospital de Leon with registration number 2304 (January 31, 2023).

## Conflicts of Interest

The authors declare no conflict of interest.

## Supporting information




**Supporting File 1**: phn70086‐sup‐0001‐SuppMat.pdf

## Data Availability

The data that support the findings of this study are available from the corresponding author upon reasonable request.
