# Supplementary material for: Exposure to Tobacco Smoke and Beliefs About Smoking Among Nursing Students and Professionals in Spain
Source: Public Health Nurs. 2026 Feb 13;43(3):564–74. doi: 10.1111/phn.70086 (PMC13108633; doi:10.1111/phn.70086)
Supplement: Supplementary file 1 — Supporting File 1: phn70086‐sup‐0001‐SuppMat.pdf [file PHN-43-564-s001.pdf]

2022 TOBACCO USE QUESTIONNAIRE - STUDENTS

Survey date: ___/___/______

PERSONAL DATA

Sex: Man ☐ Woman ☐ Degree: Nursing ☐ Physiotherapy ☐ Other ☐ ________

Date of birth: ___/___/______ University: ____________

Year: First ☐ Second ☐ Third ☐ Fourth ☐

Previous studies granting access to the degree:

High School ☐ Vocational Training ☐ Over 25 ☐ Diploma ☐ Bachelor's ☐ Graduate ☐

1. Which of the following descriptions best fits your current tobacco or electronic cigarette use?

- Non-smoker who has never smoked ______________________________ ☐ 1
- Non-smoker who has quit ______________________________________ ☐ 2
- Smoker who does not want to quit ______________________________ ☐ 3
- Smoker willing to make a serious quit attempt ____________________ ☐ 4
- Vaper (electronic cigarette) ____________________________________ ☐ 5
- Smoker and vaper ____ ☐ 6
- Non-vaper who has quit _______________________________________ ☐ 7

1a. Do any of the people you live with currently smoke? Yes ☐ No ☐

1b. Have you been exposed to smoke-filled environments in the last few days? Yes ☐ No ☐

2. In general, as a future healthcare professional:

• To what extent are you concerned about the risks and consequences of tobacco use on health?

Very much ☐ Quite ☐ A little ☐ Not at all ☐

• And about the harmful effects that secondhand smoke may have on non-smokers?

Very much ☐ Quite ☐ A little ☐ Not at all ☐

3. Has your doctor/nurse ever recommended that you quit smoking (if you are a smoker) or continue not smoking (if you are a non-smoker)?

• Always ☐ Never ☐ Sometimes ☐ They do not know I smoke ☐

4. Below is a list of health problems. For each one, indicate the relationship you believe exists between tobacco use and the problem:

**Problem**	**Main cause**	**Contributing cause**	**No relation**	**Unknown**
Lung cancer	☐	☐	☐	☐
Chronic bronchitis	☐	☐	☐	☐
Pulmonary emphysema	☐	☐	☐	☐
Throat cancer	☐	☐	☐	☐
Peripheral vascular disease	☐	☐	☐	☐
Bladder cancer	☐	☐	☐	☐
Coronary disease	☐	☐	☐	☐
Laryngeal cancer	☐	☐	☐	☐
Oral cavity leukoplakia	☐	☐	☐	☐

5. Below are some reasons that may be important for a future health professional to quit smoking. For each one, please indicate how important it is to you:

	**Very important**	**Quite important**	**Slightly important**	**Not important**
Protect health	☐	☐	☐	☐
Avoid symptoms/illnesses related to tobacco use	☐	☐	☐	☐
Pressure from other healthcare professionals	☐	☐	☐	☐
Set a good example	☐	☐	☐	☐
Avoid bothering people nearby	☐	☐	☐	☐
Avoid smoke-filled environments	☐	☐	☐	☐

6. During your clinical practice/daily life, do you encourage patients/relatives/friends to respect smoking bans?

Yes, usually ☐ Yes, occasionally ☐ No, never ☐

7. Considering the following situations, how often would you warn these patients about the risks and consequences of tobacco use?

**Situation**	**Always**	**Often**	**Sometimes**	**Rarely**	**Never**
Smoker with symptoms or diagnosed disease related to tobacco use	☐	☐	☐	☐	☐
Smoker with worsening of a previous condition not related to tobacco use	☐	☐	☐	☐	☐
Patient who directly and voluntarily asks about the consequences of tobacco use	☐	☐	☐	☐	☐

8. For each of the following health problems, indicate the relationship you believe exists between exposure to secondhand smoke/passive smoking and the problem:

**Problem**	**Main cause**	**One more cause**	**No relationship**	**Unknown relationship**
Lung cancer	☐	☐	☐	☐
Respiratory diseases	☐	☐	☐	☐
Cardiovascular diseases	☐	☐	☐	☐
Childhood asthma	☐	☐	☐	☐
Other respiratory problems in children (bronchitis, pneumonia…)	☐	☐	☐	☐
Low birth weight	☐	☐	☐	☐

9. Below are statements about the role of healthcare professionals in preventing smoking. Indicate your level of agreement:

**Statement**	**Strongly agree**	**Agree**	**Slightly agree**	**Disagree**
Health professionals' advice is very important to help and convince someone to quit smoking.	☐	☐	☐	☐
Health professionals should never smoke in front of patients to set a good example.	☐	☐	☐	☐
Health professionals serve as social role models regarding smoking.	☐	☐	☐	☐

Smokers pay close attention to health professionals' recommendations about tobacco's harmful effects.	☐	☐	☐	☐
Health professionals have an obligation to try to convince patients to quit smoking.	☐	☐	☐	☐

10. Indicate your degree of agreement with the following statements regarding the training and actions of healthcare professionals:

**Statement**	**Strongly Agree**	**Agree**	**Slightly Agree**	**Disagree**
My knowledge allows me to provide accurate information about the consequences of tobacco use.	☐	☐	☐	☐
I currently have enough knowledge to effectively help a patient quit smoking.	☐	☐	☐	☐
Students must receive specific training to help patients quit smoking.	☐	☐	☐	☐
Topics related to smoking prevention should be included in training.	☐	☐	☐	☐
Health professionals play a very important social role in preventing tobacco use.	☐	☐	☐	☐
I know strategies and methods to help patients quit smoking.	☐	☐	☐	☐

Regarding substance use:

1. I do not use any substances ☐

2. I use (check all that apply): Tobacco ☐ Alcohol ☐ Marijuana/hashish ☐ Cocaine ☐ Ecstasy/MDMA ☐ Methamphetamine/crystal ☐ Heroin ☐ Mushrooms ☐

IF YOU ARE A NON-SMOKER YOU MAY SUBMIT THE QUESTIONNAIRE NOW. THANK YOU FOR YOUR COLLABORATION.

IF YOU ARE A FORMER SMOKER, GO TO QUESTION 11

IF YOU ARE A SMOKER, GO TO QUESTION 14

FORMER SMOKERS

11. At what age did you start smoking? ___ years

12. How long ago did you quit smoking? ___ days

13. What were the two main reasons that led you to quit smoking?

Tobacco harmed my health ☐ Economic reasons ☐ Because it was a dirty habit ☐

Tobacco was harmful to my health ☐ Bad example ☐ Family pressure ☐

Tobacco harms others' health ☐ I did not want tobacco to control me ☐ Other ☐ ______

MAY SUBMIT THE QUESTIONNAIRE NOW. THANK YOU FOR YOUR COLLABORATION.

SMOKERS

14. At what age did you try your first cigarette? ___

15. At what age did you begin smoking regularly? ___

16. How many years have you been smoking? ___

17. Why did you start smoking? (main reason)

Most of my friends smoked ☐

My best friend (someone important to me) smoked ☐

I felt older, independent ☐

To feel more included ☐

I liked the taste and smell ☐

Other (specify): ___________

17a. Regarding cigarette consumption:

17a1. If you are a daily smoker, how many cigarettes do you smoke a day? ___ cig/day

17a2. If you are an occasional smoker, indicate the option that best fits your situation:

a) ___ cig/week b) ___ cig/month

18. You currently smoke cigarettes:

Entirely to the filter ☐ Almost the whole cigarette ☐ Half the cigarette ☐ Light it and put it out ☐

19. Do any of the people you live with currently smoke?

Yes ☐ No ☐

20. If yes, who?

Father ☐ Mother ☐ Partner ☐ Roommate ☐ Others ☐

21. Do you consider your group of friends to be smokers?

Yes ☐ No ☐

22. Did you start smoking at university?

Yes ☐ No ☐

23. TEST

How many cigarettes do you smoke per day? 1–10 ☐ 11–20 ☐ 21–30 ☐ ≥31 ☐

How long after waking do you smoke your first cigarette?

Less than 5 min ☐ 6–30 min ☐ 31–60 min ☐ More than 60 min ☐

Do you smoke in the morning? Yes ☐ No ☐

Do you find it difficult not to smoke in places where it is forbidden? Yes ☐ No ☐

Do you smoke when you are sick? Yes ☐ No ☐

Which cigarette gives you the most satisfaction? First of the day ☐ Others ☐

24. TEST

Would you like to quit smoking? No ☐ Yes ☐

How interested are you in quitting? (0 not at all – 3 very seriously): 0 / 1 / 2 / 3

Would you try to quit in the next few weeks? (0 definitely NO – 3 definitely YES): 0 / 1 / 2 / 3

What is the likelihood that you will be a former smoker in six months? (0 definitely NO – 3 definitely YES): 0 / 1 / 2 / 3

YOU MAY SUBMIT THE QUESTIONNAIRE NOW. THANK YOU FOR YOUR COLLABORATION.

2022 TOBACCO USE QUESTIONNAIRE – HOSPITAL

Survey date: ___/___/______

PERSONAL DATA

Sex: Man ☐ Woman ☐ Date of birth: ___/___/______

Professional experience in years: 0–5 years ☐ 5–10 years ☐ 10–15 years ☐ More than 15 years ☐

Work area:

Medical ward ☐ Surgical ward ☐ Outpatient clinics ☐

Operating room/Anesthesia ☐ Special services (ICU, ER, Dialysis…) ☐ Pediatrics ☐ Other ☐

Type of contract:

Permanent ☐ Interim ☐ Temporary ☐

1. Which of the following descriptions best fits your current tobacco or electronic cigarette use?

• Non-smoker who has never smoked ______________________________ ☐ 1

• Non-smoker who has quit _____________________________________ ☐ 2

• Smoker who does not want to quit _______________________________ ☐ 3

• Smoker willing to make a serious quit attempt ________________________ ☐ 4

• Vaper (electronic cigarette) ____________________________________ ☐ 5

• Smoker and vaper ____ ☐ 6

• Non-vaper who has quit ________________________________ ☐ 7

1a. Do any of the people you live with currently smoke? Yes ☐ No ☐

1b. Have you been exposed to smoke-filled environments in the last few days? Yes ☐ No ☐

2. In general, as a healthcare professional:

• To what extent are you concerned about the risks and consequences of tobacco use on health?

Very much ☐ Quite ☐ A little ☐ Not at all ☐

• And about the harmful effects that secondhand smoke may have on non-smokers?

Very much ☐ Quite ☐ A little ☐ Not at all ☐

3. Has your doctor/nurse ever recommended that you quit smoking (if you are a smoker) or continue not smoking (if you are a non-smoker)?

• Always ☐ Never ☐ Sometimes ☐ They do not know I smoke ☐

4. Below is a list of health problems. For each one, indicate the relationship you believe exists between tobacco use and the problem:

**Problem**	**Main cause**	**Contributing cause**	**No relation**	**Unknown**
Lung cancer	☐	☐	☐	☐
Chronic bronchitis	☐	☐	☐	☐
Pulmonary emphysema	☐	☐	☐	☐
Throat cancer	☐	☐	☐	☐
Peripheral vascular disease	☐	☐	☐	☐
Bladder cancer	☐	☐	☐	☐
Coronary disease	☐	☐	☐	☐
Laryngeal cancer	☐	☐	☐	☐
Oral cavity leukoplakia	☐	☐	☐	☐

5. Below are some reasons that may be important for a healthcare professional to quit smoking. For each one, please indicate how important it is to you:

Reason	Very important	Quite important	Little importance	Not important
Protect health	☐	☐	☐	☐
Avoid symptoms/illnesses related to tobacco use	☐	☐	☐	☐
Pressure from other healthcare professionals	☐	☐	☐	☐
Set a good example	☐	☐	☐	☐
Avoid bothering people nearby	☐	☐	☐	☐
Avoid smoke-filled environments	☐	☐	☐	☐

6. During your workday/daily life, do you encourage patients/relatives/friends to respect smoking bans?

Yes, usually ☐ Yes, occasionally ☐ No, never ☐

7. Considering the following situations, how often would you warn these patients about the risks and consequences of tobacco use?

Situation	Always	Often	Sometimes	Rarely	Never
Smoker with symptoms or diagnosed disease related to tobacco use.	☐	☐	☐	☐	☐
Smoker with worsening of a previous condition not related to tobacco use.	☐	☐	☐	☐	☐
Patient who directly and voluntarily asks about the consequences of tobacco use.	☐	☐	☐	☐	☐

8. For each of the following health problems, indicate the relationship you believe exists between exposure to secondhand smoke/passive smoking and the problem:

**Problem**	**Main cause**	**One more cause**	**No relationship**	**I don't know the relationship**
Lung cancer	☐	☐	☐	☐
Respiratory diseases	☐	☐	☐	☐
Cardiovascular diseases	☐	☐	☐	☐
Childhood asthma	☐	☐	☐	☐
Other respiratory problems in children (bronchitis, pneumonia…)	☐	☐	☐	☐
Low birth weight	☐	☐	☐	☐

9. Below are statements about the role of healthcare professionals in preventing smoking. Indicate your level of agreement:

**Statement**	**Strongly agree**	**Agree**	**Slightly agree**	**Disagree**
Health professionals' advice is very important to help and convince someone to quit smoking.	☐	☐	☐	☐
Health professionals should never smoke in front of patients to set a good example.	☐	☐	☐	☐
Health professionals serve as social role models regarding smoking.	☐	☐	☐	☐
Smokers pay close attention to health professionals' recommendations about tobacco's harmful effects.	☐	☐	☐	☐
Health professionals have an obligation to try to convince patients to quit smoking.	☐	☐	☐	☐

10. Indicate your degree of agreement with the following statements regarding the training and actions of healthcare professionals:

**Statement**	**Strongly Agree**	**Agree**	**Disagree**	**Strongly Disagree**
My knowledge allows me to provide accurate information about the consequences of tobacco use.	☐	☐	☐	☐
I currently have enough knowledge to effectively help a patient quit smoking.	☐	☐	☐	☐
Students must receive specific training to help patients quit smoking.	☐	☐	☐	☐
Topics related to smoking prevention should be included in training.	☐	☐	☐	☐
Health professionals play a very important social role in preventing tobacco use.	☐	☐	☐	☐
I know strategies and methods to help patients quit smoking.	☐	☐	☐	☐

Regarding substance use:

1. I do not use any substances ☐

2. I use (check all that apply): Tobacco ☐ Alcohol ☐ Marijuana/hashish ☐ Cocaine ☐ Ecstasy/MDMA ☐ Methamphetamine/crystal ☐ Heroin ☐ Mushrooms ☐

IF YOU ARE A NON-SMOKER YOU MAY SUBMIT THE QUESTIONNAIRE NOW. THANK YOU FOR YOUR COLLABORATION.

IF YOU ARE A FORMER SMOKER, GO TO QUESTION 11

IF YOU ARE A SMOKER, GO TO QUESTION 14

FORMER SMOKER

11. At what age did you start smoking? ___ years

12. How long ago did you quit smoking? ___ days

13. What were the two main reasons that led you to quit smoking?

Tobacco harmed my health ☐ Economic reasons ☐ Because it was a dirty habit ☐

Tobacco was harmful to my health ☐ Bad example ☐ Family pressure ☐

Tobacco harms others' health ☐ I did not want tobacco to control me ☐ Other ☐ ______

MAY SUBMIT THE QUESTIONNAIRE NOW. THANK YOU FOR YOUR COLLABORATION.

SMOKERS

14. At what age did you try your first cigarette? ___

15. At what age did you begin smoking regularly? ___

16. How many years have you been smoking? ___

17. Why did you start smoking? (main reason)

Most of my friends smoked ☐

My best friend (someone important to me) smoked ☐

I felt older, independent ☐

To feel more included ☐

I liked the taste and smell ☐

Other (specify): ___________

17a. Regarding cigarette consumption:

17a1. If you are a daily smoker, how many cigarettes do you smoke a day? ___ cig/day

17a2. If you are an occasional smoker, indicate the option that best fits your situation:

a) ___ cig/week   b) ___ cig/month

18. You currently smoke cigarettes:

Entirely to the filter ☐ Almost the whole cigarette ☐ Half the cigarette ☐ Light it and put it out ☐

19. Do any of the people you live with currently smoke?

Yes ☐ No ☐

20. If yes, who?

Father ☐ Mother ☐ Partner ☐ Roommate ☐ Others ☐

21. Do you consider your group of friends to be smokers?

Yes ☐ No ☐

22. Did you start smoking at university?

Yes ☐ No ☐

23. TEST

How many cigarettes do you smoke per day? 1–10 ☐ 11–20 ☐ 21–30 ☐ ≥31 ☐

How long after waking do you smoke your first cigarette?

Less than 5 min ☐ 6–30 min ☐ 31–60 min ☐ More than 60 min ☐

Do you smoke in the morning? Yes ☐ No ☐

Do you find it difficult not to smoke in places where it is forbidden? Yes ☐ No ☐

Do you smoke when you are sick? Yes ☐ No ☐

Which cigarette gives you the most satisfaction? First of the day ☐ Others ☐

24. TEST

Would you like to quit smoking? No ☐ Yes ☐

How interested are you in quitting? (0 not at all – 3 very seriously): 0 / 1 / 2 / 3

Would you try to quit in the next few weeks? (0 definitely NO – 3 definitely YES): 0 / 1 / 2 / 3

What is the likelihood that you will be a former smoker in six months? (0 definitely NO – 3 definitely YES): 0 / 1 / 2 / 3

MAY SUBMIT THE QUESTIONNAIRE NOW. THANK YOU FOR YOUR COLLABORATION.
